# The Continued Use of the Antiseptic and Eliminative Treatment of Typhoid Fever without Any Deaths

**Published:** 1900-08

**Authors:** T. Virgil Hubbard

**Affiliations:** Atlanta, Ga.


### THE CONTINUED USE OF THE ANTISEPTIC AND ELIMINATIVE TREATMENT OF TYPHOID FEVER WITHOUT ANY DEATHS.*

BY T. VIRGIL HUBBARD, M.D.,

ATLANTA, GA.

Continued and uninterrupted success with the antiseptic and eliminative treatment, with convincing clinical illustrations of its inevitable correctness, forces me to disregard the sage advice of Shakespeare, and repeat some remarks I made before this Association one year ago. Assuming the risk of being ridiculed for repeating the truth, and perhaps denounced for making it so emphatic, I acknowledge my cheerfulness to submit to either, if by so doing I may induce some of my professional friends to cast aside their theoretical objections, which are too often but the delusions of a dream which can be swiftly dispelled by the awakening light of clinical experience with the treatment.

With profound respect for those gentlemen (for some of them I love) who differ with me from a theoretical standpoint, I must confess a failure to see the logic in attempting to combat clinical results with theoretical argument; and especially when that argument is based on an acknowledged inexperience with this treatment and is influenced and sustained by the passion and prejudice naturally engendered by following for years a contrary course of treatment. If medicine were an exact science, if we could demonstrate the correctness or incorrectness of its theories as we can a mathematical problem, there would then be some logic in resorting to mere argument to meet the mortality statistics; but at the present time the court that renders the final decision, and from which there can be no appeal, is the number of deaths which follow from this or

*Read before the Georgia Medical Association, April, 1900.

that line of treatment. That we are all creatures of habit and routine is unfortunately too true, and medical men are no exception to the general rule. A study of the history of medicine reveals the fact that all new theories or advance in treatment undergo a struggle for existence in their infancy, and the friends of this treatment are not in the least discouraged because it is undergoing the fire of denunciation and criticism which characterizes a radical change of any established treatment. It would be nothing short of a miracle if the medical profession should spontaneously adopt the eliminative treatment, which is almost the opposite of what has been taught and practiced for the past twenty-five years, regardless of its correctness or incorrectness. A child reared in a Protestant family, and who has been taught the edicts of that religion, is not likely to embrace Roman Catholicism, and *vice versa.* So the young man who is taught in his college course that cold water is the proper treatment for typhoid fever, and has practiced it for years after leaving his *alma mater,* is not likely to adopt the eliminative treatment without indubitable evidence of its correctness.

I will briefly repeat the treatment which I recommended in a paper read before this Association one year ago, and I have very few additions to make to the same. When called to a case of typhoid fever, I usually commence by giving the patient a capsule of calomel $\frac{1}{2}$ grain, guaiacol carbonate 2 grains, podophyllin 1-20 to 1-40 grain, every two hours for twenty-four to forty-eight hours, depending upon the condition of the bowels. I continue this until I have secured four or five intestinal evacuations for two successive days, and then I leave off the calomel and add $\frac{1}{2}$ grain of menthol to the guaiacol and podophyllin. If after discontinuing the calomel there is any tendency (as there frequently is) of the bowels to become inactive, I administer a small dose of salts or Hunyadi water in the morning. I always endeavor to secure at least two or more evacuations daily, depending upon the temperature and the condition of the bowels. If, after four or five days of treatment, the temperature remains high or rises after having remained stationary, I again resort to the calomel as before for twenty-four hours or less, as necessary, and it invariably reduces the temperature and results in a general improvement in the patient's condition. I continue the administration of guaiacol and menthol throughout the course of the disease.

In addition to this I frequently resort to the administration of normal salt solution per rectum, and have been very much gratified with its results, especially in those cases where the skin and kidneys failed to act well. It seems to me that in any toxemia normal saline solution would do good by diluting the poison in the blood and favoring its elimination by the skin and kidneys; and in case of very high fever or severe toxemia and an exhausted condition of the patient, I would promptly resort to its subcutaneous administration.

I will consider first the least important, the *antiseptic* part of the treatment. It has been said by the opponents of this treatment that it was impossible to render the alimentary canal *aseptic* by the administration of drugs (an opinion in which I have always heartily concurred); but I was surprised a year ago to find opposition to this treatment from some of our surgeons who practise aseptic technique in their operations, for if they will reflect but for a moment, it is this same broad principle which, applied to surgery, has rescued it from a chaotic state of empiricism and elevated it to a scientific basis. Would any modern surgeon fail to wash out and drain an abscess cavity because he could not render it aseptic at the first operation? Should he fail to irrigate an infected wound because he cannot cleanse it of every drop of pus and every micro-organism at the first irrigation? If a patient is bleeding from three ruptured arteries, and from an anatomical situation one of them cannot be reached, does it lessen the obligation of the surgeon to place a ligature around the other two? So in typhoid fever, if we cannot thoroughly disinfect the bowels and thus reach ideal results, must we peacefully fold our hands and acknowledge with mortification and chagrin that we can do nothing but amuse the patient by pouring cold water upon the skin? Is this the course the surgeon pursues in a case of infection? Is it not our plain and unequivocal duty, with our present knowledge of causation and pathology of typhoid fever, to resort to those measures, first, that will remove so far as possible the offending agent *from* the bowels? and, secondly, render the intestine, so far as possible, an unfit culture tube for multiplication and development of the various micro-organisms which are to be found there, as well as to prevent fermentation and putrefaction of food products? Is not the efficacy which has for a long time been attributed to that good old remedy turpentine been due almost exclusively to its antiseptic qualities? But whatever may be said in favor of the administration of intestinal antiseptics in typhoid fever, they play an insignificant part as compared with those remedies which have for their function the promotion of *elimination* from the system of the toxins and by stimulation of the emunctory organs. It is to the accomplishment of this result that the successful clinician will chiefly address his efforts.

To those who have attempted to refute something I have never claimed in reference to intestinal antiseptics, I would most respectfully invite their attention to the eliminative part of the treatment. I presume no one is so obsolete in his views as to maintain for a moment that the intestinal ulceration, *per se*, in the bowel wall is the cause cf the constitutional symptoms of typhoid fever. If so, how does he account for those fatal cases of typhoid in which no intestinal lesion is found post mortem? The most convincing fact gleaned from my experience with the eliminative treatment is that entirely *too much importance* has heretofore been attached to the *intestinal ulceration* and too little to the *condition of the patient.* The sins of omission are too numerous to mention which will be justly heaped upon that timid physician who sits by a case of typhoid waiting and expecting perforation of the bowel or fatal hemorrhage while assuming an attitude of passive indifference; fearing something which, in the large majority of cases, will never happen, and on this account withholding both food and drugs while the life of his patient is surely but slowly ebbing away from starvation and toxemia. That a healthful reaction has set in against a too restricted diet in typhoid fever no one can deny who is familiar with recent articles in the leading medical journals. I am convinced that a large number of typhoid patients have died from want of food, and it is equally true that a great many have passed to the "Beyond" because they were unable to digest and assimilate what was given them, owing to the torpid condition of the liver and diminished secretion of gastric and intestinal juices, which could be largely overcome by the administration of proper drugs. The ulceration of the bowel has been the great *bugbear* of the physician—the objective point on which he has centered his therapeutic vision—and if it does not produce perforation or a fatal hemorrhage, it is of no more prognostic significance than if it were on the leg of the patient, barring the exception that it may act as an absorbing surface for other germs than the bacillus of Eberth. While the attention of the physician has thus been directed to the condition of the bowel and temporarily distracted by the fear of a perforation or a fatal hemorrhage, which only occurs in about two or three per cent. of the cases, he has wilfully and persistently neglected those measures at his command which would prevent the death of his patient from exhaustion and toxemia. Some old writer has said that the physician should always obviate the tendency to death; and we can sometimes learn more from one case that dies than from ten cases that recover by studying the cause of the death. I think we will gain some practical information by closely observing the cause of the tendency to death and become more active in the use of those remedies that combat that tendency. The sooner we look upon typhoid fever as a constitutional poisoning of the system very similar to an average case of septicemia, and treat it accordingly by assisting nature to eliminate the poison or destroy it in the system, the sooner we will diminish the mortality of this disease.

There are certain proven facts about this disease on which I think all will agree and will accept:

1. *That the cause of typhoid fever does not originate, de novo, in the human organism, consequently it must be introduced from without.*

2. *That this causative agent introduced from without and the poisonous products it generates within come under the definition of constitutional poisoning, and may be found in almost every tissue of the body.*

3. *That at the present time we do not possess a direct chemical antidote whereby this poison may be changed into a harmless compound.*

With these propositions admitted—a poison introduced into the system, circulating in the blood and found in the different tissues of the body, and for which we have no chemical antidote—our duty becomes plain and the therapeutics of this disease very much simplified. What would we do in other cases of poisoning with something for which we had no direct antidote? Would we not attempt to eliminate it from the system? The rational procedure, then, is the elimination of this poison from the system; and its egress can only be accomplished by stimulating to the fullest functional capacity the liver, bowels, kidneys, and skin—the natural eliminants. That nature, when left alone, attempts to throw off the poison by these organs is clearly shown by the fact that their secretions contain the lethal products and will convey the disease. Futerer found that bacteria injected into the portal vein appeared in the general circulation in one minute, and he also demonstrated that elimination of the germ from the system by the liver and kidneys was very promptly inaugurated. He suggests that this eliminative function of the liver explains why the typhoid germ is so often found in the gall-bladder and bile-ducts, where it frequently sets up an inflammatory process.

Adami has also demonstrated that the liver cells normally possess a bactericidal function aside from that of elimination.

With these facts before us, I think we should apply those remedies which, from a knowledge of their physiological effect, will most actively and harmlessly assist nature in this eliminative process. While opinions may differ as to the efficacy of this or that drug, there can be no question as to the object to be accomplished. Those who practice and teach drug nihilism in reference to the treatment of typhoid fever should recognize the fact that their sins of omission are sometimes greater than those of commission, and that there are other pitfalls in the pathway of the physician than those of mischievous activity in the administration of drugs. The innocuous desuetude into which some teachers and practitioners of medicine have lapsed in reference to the drug treatment of infectious diseases, and especially typhoid fever, and the common acceptance of the purely imaginary theory that because a disease is produced by a germ it must necessarily run a definite and specific course, uninfluenced by any remedial measures, is, to my mind, the most rational explanation of why so many laymen are to-day seeking relief at the hands of the water-cure man, the Christian Scientist and osteopathist—glaring illustrations of which we have in our own city. The advice given by a prominent text-writer in this country, that the physician, in managing a case of typhoid fever, should assume an attitude of "armed expectancy," it seems to me, if strictly adhered to, would be the indirect cause of more deaths from this disease than any advice which might be couched in any other *two* words in the English language. Aanalyze those words, what do they imply? They mean that the physician should nurse the patient and wait for complications, thus shattering to smithereens the good old doctrine that an ounce of prevention is worth a pound of cure. Of what practical value is the most up-to-date and thoroughly equipped therapeutic armamentarium in the presence of perforation of the bowel or a severe hemorrhage in typhoid fever? Even though the physician expected it to occur, even though he be prepared with all known measures to meet the condition, of what practical value is it to the patient when he almost invariably passes to that land from whose bourne no traveler returns, and leaves the physician in a state of melancholy reflection on the inadequacy of his "armed expectancy"?

I think it wiser to use those measures which, in a large number of instances, will prevent the complication than it is to await their development and then attempt to repair their ravages. That complications can be prevented by the administration of the proper remedies I have conclusively demonstrated by my own clinical results. In selecting those remedies which will most effectively promote elimination, we naturally place mercury at the head as the great glandular stimulant. That it is justly entitled to the first place as a secretory and excretory stimulant years of experience have conclusively proven. It is immaterial into what remote part of the system or what complex tissue the typhoid germ or its toxin may migrate; that it cannot evade the presence of this protean drug is shown by the fact that the secretion from the testicle of the male and the mammary gland of the female are no exception to the ubiquitous presence of this drug. Those who have never used mercury throughout the course of typhoid fever, and have objected to it on the grounds that it was exhausting and tended to weaken the patient, entirely overlook the fact that it is a tonic in small doses in health and disease. Cabot and others have demonstrated that it will increase the number of red blood-corpuscles and the hemaglobin and augment the body-weight in small doses. There is practically no drug of value which, in the hands of the careless or ignorant, would not do harm. Quinine is a specific in malarial fever, mercury is a specific for syphilis; but who will deny that you can kill a malarial patient with quinine and a syphilitic with mercury? But does this lessen their value when properly, carefully and scientifically administered in these diseases?

It is to the proper administration of calomel in typhoid fever that I desire especially to call attention. If those gentlemen who are sceptical regarding the efficacy of this treatment will administer the drug in small and oft-repeated doses, carefully watching for its constitutional effect, they will find that it will control the unpleasant symptoms and fatal tendency of this disease. While it has this effect in small doses, yet to give it in five- to seven-grain doses three times a day would be as irrational and as open to criticism as to start a syphilitic on the same large dose. I have conclusively proven that a typhoid patient can take more mercury without purgation or salivation than the same individual can take when not suffering from the disease. I think we may attribute some of the good effects of mercury in typhoid to the stimulation of the production of white blood-cells, which are the great protectors of the system in all infectious processes. While I cannot state this positively, because I have not counted the cells while a typhoid patient was taking mercury, yet I think we are justified in assuming this on theoretical grounds.

I have, up to the present time, treated twenty cases of typhoid by this method without any deaths; other men have treated a larger number of cases without any deaths, and the collected results give a mortality of less than two per cent. The erroneous position of those who oppose this treatment, and at the same time admit that they have never used it, is too conspicuous to require more than passing notice. If our friends will resort to the same unprejudiced investigation and seek that same high class of indubitable evidence—clinical results—which has characterized our investigation of the subject, they will then be competent witnesses, and we will welcome with enthusiasm a report of their results. When an individual or scientific body of investigators desires to determine the efficacy of any therapeutic measure, do they seek the opinion of physicians who, from want of experience with that measure, are unfamiliar with its results? or do they rather inquire of those men who, having had clinical experience with the use of the measure under consideration, are qualified to give testimony? If a physician treat a given number of cases of appendicitis medicinally and get a mortality of about ten per cent., by what rule of logic would he be justly entitled to condemn the measure of a surgeon who operated on an equal number of cases and got a mortality of two per cent.?

The physician who has never witnessed the contrast in the clinical picture of a typhoid patient on the eliminative treatment as compared to the expectant plan can no more appreciate its efficacy than a person born blind can appreciate the beauties of nature. If he has never witnessed the prompt disappearance of delirium, the restoration of consciousness to the clouded intellect, prompt return of the appetite and digestive capacity, and gradual but sure reduction of temperature, he has in store for himself one of the triumphs of his profession and a scene which will give him renewed confidence in the healing art. It is the truth we are seeking in this matter, regardless of the source from which it sprang; and if our opponents will show by clinical experience with this treatment that the mortality is as great or greater than any other method now known, we will then, *but not till then,* cheerfully acknowledge our error.

*210 Fitten Building.*

